# Total hip arthroplasty: 30 days readmission at a tertiary care hospital

**DOI:** 10.12669/pjms.41.4.11041

**Published:** 2025-04

**Authors:** Muhammad Omer Farooq, Mahnoor Tariq, Ahsan Sulaiman, Shahryar Noordin

**Affiliations:** 1 Muhammad Omer Farooq, MBBS Resident, Section of Orthopedic Surgery, The Aga Khan University, Karachi, Pakistan; 2 Mahnoor Tariq, MBBS; MSc Research Specialist, Department of Paediatrics & Child Health, The Aga Khan University, Karachi, Pakistan; 3 Ahsan Sulaiman, MBBS, The Aga Khan University, Karachi, Pakistan; 4 Shahryar Noordin, M.B.B.S. (AKU), FCPS, FACS, FRCS (Glasg), FRCS (Eng), The Aga Khan University, Karachi, Pakistan

**Keywords:** Arthroplasty, Hip Replacement, Patient Readmission, Tertiary Care Centers

## Abstract

**Objectives:**

To assess 30-day readmission at a tertiary care hospital following Total Hip Arthroplasty (THA) and to assess complications for which the patients were readmitted along with associated factors.

**Methods:**

This prospective observational study examined patients undergoing THA at The Aga Khan University Hospital from February 2023 to December 2023. Patients were followed after THA up to 30 days. They were followed using an electronic medical record number system. Data on patient demographics and procedure was collected, along with reason for re-admission within 30-days. Statistical analysis was done using STATA version 15.1, where in descriptive statistics median and interquartile range, and frequencies and percentages were determined followed by Fisher’s exact test.

**Results:**

A total of 67 patients were included in the study. Four male patients above 55 years were readmitted within 30-days of THA. Underlying diagnosis was a significant factor for re-admission (p=0.05). Although, OA and AVN were commonly reported in patients >55 years, two of the readmitted patients had an underlying diagnosis of femoral neck fracture.

**Conclusion:**

Diagnosis at the time of admission for THA is a substantial risk factor for readmission that warrants further investigation. In addition, it underscores the need for targeted post-operative monitoring to reduce readmissions especially for patients who are at high risk of readmission.

## INTRODUCTION

Total hip arthroplasty is one of the most successful and commonly performed procedures in orthopedic surgery. THA provides a cost-effective intervention for end stage hip arthritis offering pain free mobility, improved quality of life with early return to function.^1^-^5^ Additionally, THA with enhanced bearing surfaces and surgical techniques have been associated with favorable long term outcomes with implant survival rates exceeding 90% after 15 to 25 years.^6^,^7^

Internationally, over one million THAs are performed annually. The U.S recorded nearly 370,000 primary hip replacements in 2014^8^,^9^, while Australia and U.K reported 37,000 and 97,000 in 2017, respectively. In Pakistan, THA incidence was 2.26 per 100,000 from 2014 to 2021.

With such rise in the frequency of hip replacement, a thorough analysis of the procedure and its complications is needed to minimize both risk to patients and healthcare expenditure. This is even more important in local context as only a small percentage of patients have insurance coverage to support procedure and treatment fee. Complications can be broadly classified into general and procedure specific. General complications include wound infections, postoperative pulmonary issues and thromboembolic complications. Specific complications include dislocations, iatrogenic sciatic injury, leg length discrepancy, periprosthetic fractures and deep seated implant infection.^10^

The 30-day readmission rate following THA is an important measure to evaluate postoperative results. Studies has revealed several risk variables, such as advanced age, hypertension, steroid usage, bleeding problems, and extended hospital stays, that are linked to 30-day readmission following THA.^11^ Short-term readmission rates have been cited in various articles (ranging from 2.9% to 15.6%) after elective primary THA within 30 and 90 days.^12^ In our region there is considerable lack of literature regarding readmissions following THA. To address this gap, we evaluate 30-day readmission after THA and the associated reasons.

## METHODS

This prospective observational study was conducted at the Aga Khan University Hospital, Section of Orthopedics, from February 2023 to December 2023. Patients admitted for THA were included. Patients undergoing revision arthroplasty or those with metastatic lesions were excluded. Patients were followed for 30 days after THA for readmission. Each patient had a unique medical record number which was used to identify them, and extract their data.

### Ethical Approval:

The study protocol was approved by the by the AKU Ethical Review Committee (ERC: 2023-8297-24090; dated February 20, 2023).

Sample size was calculated using Open Epi 3.0. The minimum sample size required for this study was 35 hip arthroplasty patients considering an inflation of 40% for loss to follow-up by anticipating a frequency of 1.60%^13^ of 30 days readmission with precision of 5% and design effect of 1. We performed our analysis on STATA version 15.1. For quantitative variables we reported median and interquartile range to control for skewness and for categorical variables we reported frequencies and percentages. In addition, continuous variables including age and length of stay, body mass index was sub-grouped based on clinically relevant cut-offs for interpretation and analysis. Outcome of interest was 30-day readmission (yes/no), and the number of days since discharge was recorded for each. For inferential analysis between independent variables and outcome within 30 days we applied Fisher’s exact test for categorical variables. A p-value of <0.05 was considered as statistically significant.

## RESULTS

A total of 69 patients admitted for total hip arthroplasty during the study period were reviewed. Two patients were excluded due to diagnosis of metastatic lesion in the hip, resulting in 67 patients record available for analysis. Patient’s demographic data showed in (Table-I).

**Table-I:** Characteristics of patients admitted for hip replacement surgery.

Variable	n=67 (%)
***Age (years)***	55 (17 to 91)*
≤55	34 (50.75)
>55	33 (49.25)
***Sex***	
Male	39 (58.21)
Female	28 (41.79)
***Body Mass Index (kg/m^2^)***	25 (18 to 37.8)*
Underweight (<18.5)	4 (5.97)
Normal (18.5 – 24.9)	28 (41.79)
Overweight (25 – 29.9)	27 (40.30)
Obese (≥ 30)	8 (11.49)
***ASA Level***	
1	14 (20.90)
2	39 (58.21)
3	14 (20.90)
***Diagnosis***	
Avascular Necrosis	27 (40.30)
Osteoarthritis	11 (16.42)
Femur Neck Fracture	8 (11.94)
Others	21 (31.34)
***Comorbidities***	
None	30 (44.78)
At least 1	16 (23.88)
2 or more	22 (32.84)
***Route of admission***	
Emergency	13 (19.40)
Elective	54 (80.60)
***Type of Procedure***	
Bilateral THR	8 (11.94)
Left THR	20 (29.85)
Right THR	25 (37.31)
THR with additional procedure	14 (20.90)
***Length of hospital stay (days)***	5 (4 to 20)*
≤ 5	47 (70.15)
> 5	20 (29.85)
***Blood Transfusion***	
Yes	1 (1.45)
No	66 (98.15)

* Median and interquartile range.

Within 30 days of surgery only four patients were readmitted (Table-II). All were males, with two underwent an emergency procedure while two had elective surgery. Two patients were diagnosed with femoral neck fracture and only one patient was <60 years old. Patients’ diagnosis at the time of admission reported marginal significant association (p=0.05) with 30-day readmission (Table-III). We further investigated the link between diagnosis, age and readmission status (Table-IV).

**Table-II:** Characteristics of patients readmitted after THA.

No.	Age & Sex	Route of admission	Readmission day	Diagnosis	Procedure	All-cause readmission
1	55, Male	Elective	7	Bilateral hip AVN	Bilateral THA	Painaton surgical site (SI)
2	62, Male	Elective	9	Secondary hip OA	Left THA	Hematoma formation at SI
3	69, Male	Emergency	16	Femoral neck fracture	Right THA	Acute urinary retention
4	68, Male	Emergency	18	Femoral neck fracture	Right THA	MI

Total Hip Arthroplasty (THA), Avascular Necrosis (AVN), Osteoarthritis (OA).

**Table-III:** Relationship between patient characteristics and 30-day readmission after THA.

Variables	p-value*
***Age (years)***	
≤55	0.35
>55	
***Sex***	
Male	0.13
Female	
***Body Mass Index (kg/m^2^)***	
Underweight	0.71
Normal	
Overweight	
Obese	
***ASA Level***	
1	0.81
2	
3	
***Diagnosis***	
Osteoarthritis	0.05
Avascular Necrosis	
Femur Neck Fracture	
Others	
***Comorbidities***	
None	0.11
At least 1	
2 or more	
***Route of admission***	
Emergency	0.16
Elective	
***Type of Procedure***	
Bilateral THR	0.65
Left THR	
Right THR	
THR with additional procedure	
***Length of hospital stay (days)***	
≤ 5	0.57
> 5	

* Fisher Exact test was used to calculate p-value (cut- off <0.05).

**Table-IV:** Cross-tabulation of THR patients with their age group and diagnosis.

	Age Group	Diagnosis (n%)			
		AVN (n=27)	OA (n=11)	FNF (n=8)	Others (n=21)
Not readmitted (n=63)	≤55 (n=33)	19 (57.58)	4 (12.12)	2 (6.06)	8 (24.24)
	>55 (n=30)	7 (23.33)	6 (20)	4 (13.33)	13 (43.33)
Readmitted (n=4)	≤55 (n=33)	1 (100)	-	-	-
	>55 (n=30)	-	1 (33.33)	2 (66.67)	-
Table shows row-wise distribution of frequencies.					

We observed that although only one patient ≤ 55 years with AVN was readmitted, but it was still the most common diagnosis in this age group (n=19, 57.58%) even when the patient was not readmitted. Despite, OA and AVN being commonly reported in patients >55 years, two of the patients readmitted had an underlying diagnosis of femoral neck fracture. The rest of the patients were followed in the clinic one week, 14 days and one month after discharge for wound assessment and mobility. On each visit, none of the patients reported issues with wound healing and mobility. All patients were able to perform their routine activities and move with a walker.

## DISCUSSION

Assessing hospital readmission after total hip arthroplasty reflects patient’s care in hospital and quality of operative procedure as complications such as infections, prosthetic dislocation and thromboembolic events can lead to unplanned readmissions. These readmissions not only lead to economic burden on patient and hospitals but also shows significant gaps in perioperative management and post discharge care. In developed countries hospitals are penalized who have an excessive 30 day readmission.^10^ Our results showed that there were four readmissions. One patient was admitted to the ER with surgical site pain after bilateral THA, another with hematoma at surgical site, a third with urinary retention and the fourth with myocardial infarction post THA. Two of the patients readmitted had initial diagnosis of femoral neck fracture which makes diagnosis at admission being significant factor for readmission. Literature also showed reasons for undergoing hip arthroplasty plays a significant role in the likelihood of readmission. Femoral neck fracture patient experience higher complication rates than hip osteoarthritis (OA) patients due to advanced age, increased comorbidities, and stress from trauma.^14^

Overall THA done for femoral neck fracture are at higher risk of readmission (6.57%) as compared to OA (2.93%) and AVN (1.83%).^14^,^15^Surgery related complications following THA, particularly dislocation and preiprosthetic fractures have been reported; however, these were not observed in our study.^10^,^16^ Socioeconomic factors could be important since all patients discharged to home and post-operative precautions monitored by family members. Literature also suggested periprosthetic fractures increase in patients who are discharged to some other facility rather than home.^10^,^17^

Among the reasons for readmission, one was readmitted with pain on surgical site after bilateral THA. Literature also showed 5% 30 day readmission because of pain following THA.^16^ Use of nerve blocks such as pericapsular nerve block found to helpful for managing pain after THA.^18^

Second patient was readmitted with surgical site hematoma which was managed conservatively with oral antibiotics leading to successful wound healing without complications. Surgical site hematoma is known complication of THA, with reported incidence rates ranging from 0.41% to 1.7%.^19^ One study found that 0.41% of patients required surgical intervention to treat postoperative hematoma after primary THA.^20^ The reported risk factors for hematoma formation include BMI > 35, history of bleeding disorders, operative time>100 mins and use of general anesthesia.^19^ None of these factors were present in our patient except general anesthesia. However, possible reason could be history of prior surgery making dissection in THA difficult during the clearance of acetabulum. Third patient with prior history of benign prostatic hyperplasia was readmitted with acute urinary retention. According to studies, the risk factors associated with the development of postoperative urinary retention include male sex and benign prostatic hyperplasia.^21^, ^22^

A major reason for readmission was myocardial infarction (MI) in one of the patients. Patient had a prior history of ischemic heart disease. He was admitted with femoral neck fracture and distal radius fracture and underwent THA and ORIF distal radius. He was readmitted with MI and needed CABG. Studies report, 30-day MI incidence is 0.12% and higher with cardiovascular disease.^23,24^ The strength of this study is first to have been done that, to the best of our knowledge, in Pakistan and adds valuable insight for local data regarding readmission because of different social and environmental factors as compared to west. While international studies have explored readmission reasons after THA, no prior data exists from developing countries. We can recommend future multi-center studies with a large study population to better understand readmission rates in Pakistan and low-middle income healthcare setups.

### Limitations:

This study has several limitations. It is a single center approach with a limited sample size which limits the generalizability of our findings. The low number of readmitted patients precluded a more detailed analysis using regression techniques to identify specific predictors of readmission. Although we did not observe many statistically significant associations in our results, further this research can still provide critical insights into the different diagnosis and their complications associated with THA that warrant further study research.

## CONCLUSION

There were three surgical and one medical 30 days readmission after total hip replacement with diagnosis at admission being substantial risk factor for readmission This small-scale observational study indicates a very low 30-day readmission rate after THR in a tertiary care facility. However, patients with femoral neck fractures treated with THR may have a higher risk of readmission. Thus, we recommend future multi-center studies with a large study population to better understand readmission rates in Pakistan and low-middle income healthcare setups.

### Author’s contribution:

**MOF:** Concept design, acquisition of data and writing of manuscript. Responsible for integrity of research.

**MT:** Literature search, Statistical analysis and reviewing of manuscript.

**AS:** Acquisition of data. Manuscript review. Critical analysis.

**SN:** Concept design. Reviewing and final approval of manuscript.
